# Gender-based violence and the inclusion of men in GBV prevention campaigns: perceptions of students in a university in South Africa

**DOI:** 10.3389/fsoc.2026.1743220

**Published:** 2026-07-10

**Authors:** Maboga V. I., Setwaba M. B., Maotoana M. R., Mashaba J. K., Mothapo P. M.

**Affiliations:** Department of Psychology, University of Limpopo, Sovenga, Limpopo Province, South Africa

**Keywords:** gender-based violence, prevention campaigns, male inclusion, university students, multi-gender approach

## Abstract

**Background:**

South Africa has one of the highest rates of gender-based violence (GBV) globally, disproportionately affecting women, girls, and the elderly. Despite numerous intervention efforts, GBV persists as a major social and public health concern. Prevention campaigns are central to awareness-raising, behavioural change, survivor support, and justice advocacy. Lately, attention is being given to the inclusion of men in GBV prevention efforts.

**Aim:**

To explored university students’ perceptions of GBV and the inclusion of men in GBV prevention campaigns.

**Objectives:**

To determine the perceptions of university students regarding the inclusion of men in GBV prevention campaigns, to determine their understanding of GBV and the importance of GBV prevention campaigns and the role of multi-gender approach (MGA) in the GBV prevention campaigns.

**Methodology:**

The study used a qualitative exploratory design guided by Social Learning Theory and Feminist Theory. Twelve third-year Psychology and Criminology students (7 females, 5 males), aged 22–29 years, were selected through purposive sampling. Data were collected using semi-structured interviews and analysed using thematic analysis.

**Results:**

Participants demonstrated a strong understanding of GBV and prevention campaigns, which shaped their perceptions of intervention strategies. Furthermore, participants supported the inclusion of men in GBV prevention campaigns and endorsed a multi-gender approach (MGA) as important in strengthening GBV prevention. However, inclusion was viewed as conditional, requiring careful structuring to ensure safety, trust, and the protection of women’s voices. Government-led campaigns were largely perceived as ineffective, inconsistent, and inaccessible, while civil society and community-based organisations were viewed as more responsive and impactful. Participants highlighted weak enforcement and limited accountability as key factors sustaining GBV.

**Conclusion:**

Students recognised the importance of GBV prevention campaigns but questioned their effectiveness due to implementation gaps, limited reach, and institutional weaknesses. The inclusion of men was seen as critical for transfiguring harmful gender norms and strengthening prevention efforts. The results emphasise the need for inclusive, multi-sectoral, and community-driven approaches that balance male engagement with survivor-centred safety and empowerment.

## Introduction

Gender-based violence (GBV) remains a major global public health and human-rights concern, with disproportionately high prevalence in low- and middle-income countries, including South Africa (Bloom, 2008; Britton, 2006; World Health Organization, 2019, 2022, 2023; UN Women, 2024). In South Africa, GBV continues to manifest in alarming rates of femicide, sexual violence, and intimate partner abuse, reflecting deeply entrenched structural inequalities and gendered power imbalances (Statistics South Africa, 2023; Centre for the Study of Violence and Reconciliation, 2024). Statistics South Africa (2023) reports that one in every five women suffered physical abuse from a partner in 2019. In addition, research reports that many women and girls endure sexual abuse and harassment in schools, interfering with their access to education and limiting their academic achievement (Human Rights Watch, 2018). Despite extensive policy frameworks and national interventions, GBV persists as a systemic issue requiring more inclusive and transformative prevention strategies. GBV encompasses physical, sexual, emotional, and psychological harm inflicted based on gender, often rooted in unequal power relations and socially constructed norms (World Health Organization, 2023; UN Women, 2024). Feminist scholarship conceptualises GBV as a structural phenomenon embedded in patriarchal systems that sustain women’s subordination (Connell, 2024; True, 2021). Violence against women is both a human rights issue and a development challenge with far-reaching social consequences (Onyejekwe, 2004). Furthermore, international organizations continue to emphasize the importance of policy reforms and human rights approaches in addressing violence against women (Organisation for Economic Co-operation and Development, 2023; United Nations Office of the High Commissioner, 2017; World Health Organization, 2022). At the same time, contemporary research highlights the importance of engaging men and boys in prevention efforts, particularly through gender-transformative approaches that challenge harmful masculinities and promote accountability (Gibbs et al., 2020; Casey et al., 2023; Gibbs et al., 2023). In university settings, students represent a critical population for GBV prevention, as young adulthood is a formative period for shaping gender attitudes and behaviours (Muluneh et al., 2020). Emerging evidence suggests that students’ perceptions of GBV interventions are influenced not only by awareness but also by trust in institutions, perceived inclusivity, and relevance of campaigns (Bongalo and Makhubele, 2024). This underscores the need to explore how university students perceive the inclusion of men in GBV prevention initiatives, particularly within the South African context where both the prevalence and complexity of GBV are pronounced.

## Literature review

The inclusion of men in GBV prevention campaigns has attracted more attention in recent years, with global evidence supporting gender-transformative approaches that actively engage men while challenging unequal power relations (Gibbs et al., 2020; Casey et al., 2023). Gibbs et al. (2023) and Ratele (2023) added that such approaches aim to reshape harmful masculinities and promote positive behavioural change, recognising that men play a critical role in both perpetuating and preventing violence.

Studies have demonstrated that harmful constructions of masculinity and gender inequality contribute substantially to the perpetration of violence against women (Greig, 2003; Jewkes et al., 2011; Graaff, 2021). Flood and Pease (2009) and Fulu et al. (2014) indicate that involving men in prevention initiatives can contribute to shifting social norms, increasing accountability, and fostering allyship in addressing GBV. However, emerging scholarship cautions that inclusion without serious reflection may reinforce existing gender hierarchies and inadvertently marginalise women’s voices (Connell, 2024; True, 2021). This tension reflects broader feminist concerns regarding power, representation, and the risk of re-centering male authority within spaces designed to support survivors.

Furthermore, within the South African context, research stress that men’s engagement in GBV prevention is shaped by socio-economic conditions, cultural norms, and institutional trust (Gibbs et al., 2023; HSRC and CGE, 2022). While some interventions have demonstrated success in promoting gender-equitable attitudes, challenges remain in ensuring that such initiatives are contextually relevant, sustainable, and inclusive without compromising survivor-centred principles. In South Africa, studies have highlighted the role of social norms, cultural expectations, and unequal power relations in sustaining violence (Britton, 2006; Rammbuda, 2023). These factors contribute to the normalisation of violence in some communities and influence how individuals perceive both GBV and efforts aimed at its prevention. This, therefore, highlights the importance of designing prevention campaigns that are responsive to local realities and geared towards challenging the deeply entrenched gender inequalities.

### Consequences of GBV

According to Basile et al. (2021) GBV has critical consequences, affecting victims physically, emotionally, and economically in both the short and long term. Physically, the violence can cause chronic pain, broken bones, and headaches, with pregnant women at risk of foetal injury, miscarriage, or early labour leading to psychological consequences. Unfortunately, in some instances certain incidents result in femicide, where female victims lose their lives to male perpetrators, which has become distressingly common in South Africa (World Health Organization, 2021). The psychological effects are extensive, encompassing anxiety, trauma and stressor-related disorders like Post traumatic stress disorder, mood disorders such as Major depressive disorder, and substance use disorders, often leading to alcohol or opioid abuse (Legg, 2018; Lieber, 2020). Economically, GBV costs South Africa billions annually, leading to productivity loss, medical expenses, and non-monetary impacts like lost earnings and diverted resources (KPMG, 2014). Argue that these consequences extend beyond individuals, affecting families and communities as well.

### Inclusion of men in GBV prevention

The inclusion of men in GBV prevention campaigns is a subject of debate, with dividing opinions. More studies report that involving men in GBV prevention is critical for addressing patriarchal norms and reshaping harmful masculinities (Anwana et al., 2024; Maubane-Moitsheki, 2025). According to Anwana et al. (2024) the attitudes of men toward economic empowerment and shifting gender roles also influence their responses to GBV interventions, with evidence showing that economic imbalances between men and women can reinforce resistance to gender equity initiatives. On the other hand, studies which support the inclusion of men argue that such an approach is essential for achieving gender equality and insist that men must be part of the solution if GBV is to be adequately addressed (Meer, 2012; Chitiga-Mabugu et al., 2014; Mulaudzi, 2017). It, therefore, believed that by involving men the society can challenge harmful gender norms and promote accountability, driving positive societal change. In South African campaigns such as Sonke Gender Justice and One Man Can have successfully implemented several initiatives aimed at engaging men in the fight against GBV by educating men about gender equality, challenging harmful masculinity, and promoting positive male role models (Sisonke Gender Justice, 2019).

However, Walton (2019) reports that feminist activists and scholars express differing views on the inclusion of men in anti-GBV campaigns, highlighting concerns about further marginalising women’s interests and emphasising men’s roles as primary perpetrators. Furthermore, Cornwall and Esplen (2014) indicates that there are general concerns that the involvement of men could overshadow the gender-specific issues faced by women and risks marginalising their voices and experiences. Movements like #TotalShutdown have explicitly called for men to refrain from participating in their campaigns, reflecting the tension within feminist activism regarding male involvement. An alternative viewpoint suggests a more collaborative approach to addressing these concerns. This approach according to Chitiga-Mabugu et al. (2014) advocates for forming partnerships between men’s organisations and female-led initiatives, working together as allies rather than in competition. This model promotes a multi-gender approach, where men act as supportive partners, contributing to a shared vision of ending GBV without diminishing the voices of women (Chitiga-Mabugu et al., 2014). The study draws on social learning theory and feminist theory GBV (Hooks, 2000; Jaggar, 2015) to explore these perspectives. Violence within educational spaces has also been critically examined through feminist pedagogical and legal perspectives, which highlight how institutions may reproduce gendered inequalities and fail to adequately protect vulnerable groups (Pietersen and Langeveldt, 2025).

### GBV prevention campaigns and interventions

In combating South Africa’s high rates of GBV, which President Ramaphosa has characterised as a “second pandemic” (Ramaphosa, 2020), different prevention campaigns were introduced. The campaigns are believed to play a crucial role in demanding justice for victims, protecting their rights, and striving for the global eradication of GBV. Effective GBV prevention requires an understanding of the broader social, political, and economic contexts that sustain violence, together with community-based interventions, educational initiatives, advocacy programmes, and empowerment strategies aimed at challenging harmful norms and promoting social transformation (Lokot et al., 2021; Gender Links, 2015; Moletsane et al., 2015; Olalere, 2022). One prominent campaign is the “16 Days of Activism against Gender Violence,” an international event held annually since 1991, involving activists, government leaders, students, academia, and the private sector to advocate an end to violence (UN Women, 2012). In South Africa, it is known as the “16 Days of No Violence Against Women and Children,” run by the South African national government inspired by the high rates of GBV in the country (South African Government, 2019). In addition, the “#Total Shutdown” in South Africa, was organised to raise awareness of the epidemic levels of violence against women, calling for a proactive stance from the government (Moosa, 2019). This movement emerged due to alarming statistics of increased femicide and sexual offence cases (Statistics South Africa, 2023), and it serves as a remembrance of past femicide victims like Anene Booysen and Karabo Mokoena, whose cases did not receive media attention (UN Women, 2018). Furthermore, Sisonke Gender Justice also actively fights GBV with its “Stop Gender Violence: A National Campaign,” aiming to create a fully costed, evidence-based, and comprehensive National Strategic Plan to end GBV (Sisonke Gender Justice, 2019). Recent evaluations of GBV prevention initiatives suggest that while awareness campaigns remain essential, their effectiveness is often limited without sustained community engagement and structural support (Fulu et al., 2014; Ellsberg et al., 2015). Evidence indicates that interventions are most effective when they combine policy enforcement, community mobilisation, and behavioural change strategies, rather than relying solely on symbolic or short-term awareness efforts (World Bank, 2024). This accentuates the importance of multi-sectoral approaches that integrate government, civil society, and community-level actors in addressing GBV. Furthermore, the effects of GBV extend beyond immediate physical harm, resulting in long-term psychological, social, and health consequences for survivors and communities (Smith et al., 2012; Vogelman and Eagle, 1991). In South Africa, the growing demand for survivor support services highlights the ongoing impact of violence and the need for accessible interventions (Rape Crisis Centre, 2021). Mental health professional support services play an important role in promoting recovery, social justice, and active empowerment (Van der Hoven, 2001).

### Theoretical framework

This study was guided by Social Learning Theory and Feminist Theory to understand students’ perceptions of GBV prevention campaigns and the inclusion of men in such initiatives. According to Social Learning Theory, by Albert Bandura, explains GBV as a learned behaviour reinforced through observation, repeated exposure to violence, and weak sanctions against perpetrators (Bandura, 1977; McLeod, 2016). In the South African context, poor enforcement of GBV laws and low conviction rates may contribute to the normalisation of violence (World Health Organization, 2024; Statistics South Africa, 2023). This theory helped understand participants’ concerns regarding ineffective interventions, weak accountability, and the need for behavioural change strategies involving men.

Feminist Theory views GBV as rooted in patriarchal systems that sustain gender inequality and power imbalances (Hooks, 2000; Jaggar, 2015). The theory assisted in explaining participants’ perceptions of women as the primary victims of GBV and concerns that male inclusion in prevention campaigns may overshadow women’s voices and survivor-centred spaces.

Together, these theories provided both behavioural and structural explanations of GBV and informed the interpretation of findings related to accountability, institutional responses, gendered power relations, and multi-gender prevention approaches.

## Problem statement

GBV continues to be a serious worldwide public health and human rights concern, affecting approximately one in three women worldwide (World Bank, 2018; UN Women, 2023; World Health Organization, 2024). Universally, 35% of women have experienced physical or sexual violence, with intimate partner violence accounting for 38% of female homicides. The COVID-19 pandemic further escalated this crisis, with an estimated 243 million women and girls experiencing violence during lockdown periods (Dlamini, 2021; UN Women, 2023). Unfortunately, South Africa continues to record among the highest rates of GBV comprehensively, including rape, femicide, and domestic violence (Meyiwa, 2017; Zinyemba and Hlongwana, 2022; Statistics South Africa, 2023). The overall national statistics indicate unwavering increasing levels of violence irrespective of ongoing prevention campaigns and policy interventions. Between July and September 2024 alone, over 10,000 rape cases, 957 female murders, and thousands of assault cases were reported, alongside rising violence against children (Human Rights Watch, 2025; Statistics South Africa, 2023). These patterns indicate a resolute gap between policy implementation and measurable outcomes. Although numerous GBV prevention campaigns exist, their efficacy remains dented, particularly in rural and marginalised communities with limited access to support services (Heise, 2011; UN Women, 2024). Additionally, further debates regarding the inclusion of men in GBV prevention reflect tensions between gender-transformative approaches and concerns about reinforcing patriarchal power dynamics. In this situation, limited research has investigated how university students key agents in shaping future gender norms perceive the inclusion of men in GBV prevention campaigns. This study, therefore, tackles this gap by looking into the student perceptions of male inclusion in GBV prevention efforts within a South African university context.

## Methodology

Qualitative approaches are particularly useful in GBV research because they provide in-depth insights into participants’ lived experiences and perceptions. According to Alsaigh and Coyne (2021), phenomenological research is especially valuable for exploring complex and sensitive social issues such as GBV. A qualitative exploratory research design was used to examine students’ perceptions of the inclusion of men in gender-based violence (GBV) prevention campaigns. A sample of 12 third-year Psychology and Criminology students (7 females and 5 males), aged 22–29 years, was selected through purposive sampling. This approach ensured the inclusion of information-rich participants with relevant academic exposure to GBV-related content, enabling an in-depth exploration aligned with the study objectives. The sample size was considered adequate for qualitative research, as the study prioritised depth of insight over generalisability, with data saturation was achieved after the twelfth interview, as no substantially new themes or insights emerged and participants’ responses became repetitive across the major areas of inquiry. Data were collected using the semi-structured face-to-face interviews to obtain an in-depth understanding of participants’ perceptions, feelings, and beliefs regarding men’s inclusion in GBV prevention campaigns (Mack et al., 2005; Deighton-Smith, 2014). The interview guide covered participants’ understanding of GBV, perceptions of prevention campaigns, views on government interventions, and attitudes towards male inclusion in GBV initiatives.

An interview guide was developed in alignment with the study’s research questions (What are the perceptions of third-year psychology students regarding the inclusion of men in gender-based violence prevention campaigns in South Africa? How do third-year psychology students understand the importance of gender-based violence prevention campaigns in South Africa? What are the perceptions of third-year psychology students regarding a multi-gender approach as an effective strategy for preventing gender-based violence?) to collect. The semi-structured format allowed for consistency across interviews while enabling participants to elaborate on their responses. Questions 1, 3, and 4 explored participants’ understanding of GBV prevention campaigns, their perceived effectiveness, and adequacy in raising awareness about GBV. Questions 5 and 6 examined perceptions of male inclusion in GBV prevention campaigns and attitudes of students toward feminist perspectives on male involvement, focussing on both inclusion and multi-gender approaches. Furthermore, question 2 provided contextual grounding by eliciting examples of GBV campaigns, while Question 7 allowed participants to share additional insights.

Generally, the data collection process followed a logical progression from general understanding to more reflective and evaluative questions, ensuring alignment with the study’s objectives. Data was analysed using thematic analysis as outlined by Braun and Clarke (2006). The process involved familiarisation with the data through repeated reading of transcripts, generating initial codes, developing, and identifying themes, and refining themes to ensure coherence with the dataset and alignment with the research objectives. As this was a qualitative study, no statistical analysis was conducted.

## Ethics

Ethical approval was obtained from the University of Limpopo Turfloop Research Ethics Committee (TREC) (Approval No: UL/TREC/2023/67). All procedures complied with ethical standards and the Protection of Personal Information Act (POPIA). Informed consent was obtained from all participants. Credibility was strengthened through in-depth semi-structured interviews and member checking, which verified the accuracy of participants’ perspectives. Dependability was maintained through a systematic and transparent research process, supported by a detailed audit trail of data collection and analysis procedures. Confirmability was achieved through reflexivity and the use of verbatim quotations to ground findings in participants’ accounts rather than researcher bias. Transferability was supported through rich descriptions of the research context, participants, and methodology, enabling the assessment of applicability to similar contexts.

## Results

Data from the interviews was recorded and transcribed verbatim and then used thematic analysis to code them (Braun and Clarke, 2006). Three main themes and five subthemes were identified. The interview guide covered aspects such as understanding and knowledge of GBV prevention campaigns, the importance of these campaigns, the effectiveness of government interventions, GBV knowledge and perceptions, the inclusion of men in GBV prevention (a multi-gender approach), and the contribution of government and social organisations in preventing GBV. By addressing these aspects, the study aimed to gather in-depth responses related to the above objectives and research questions.

### Demographic profile of the participants

Table 1 shows the demographic information of the participants. All participants were enrolled third-year psychology students at a university in Limpopo in South Africa. The gender profile of the participants included both males and females. As shown in the table, the number of women who participated was seven and the number of men was five. The total number of participants was 12. The gender profile is advantageous as it includes all genders.

**Table 1:** Demographic information.

Participants	Gender	Age	Degree	Year of study
Participant 1	Female	23	BA Criminology and Psychology	3
Participant 2	Female	24	BA Criminology and Psychology	3
Participant 3	Male	22	BA Criminology and Psychology	3
Participant 4	Male	26	BA Criminology and Psychology	3
Participant 5	Male	23	BA Criminology and Psychology	3
Participant 6	Female	25	BA Criminology and Psychology	3
Participant 7	Female	24	BA Criminology and Psychology	3
Participant 8	Male	25	BA Criminology and Psychology	3
Participant 9	Female	22	BA Criminology and Psychology	3
Participant 10	Female	29	BA Criminology and Psychology	3
Participant 11	Female	23	BA Criminology and Psychology	3
Participant 12	Male	24	BA Criminology and Psychology	3

### Themes and subthemes

Interview data were audio-recorded, transcribed verbatim, and analysed inductively using thematic analysis (Braun and Clarke, 2006). The three main themes and subthemes emerged as presented in Table 2 thematic structure. Themes include (a) Knowledge of GBV and prevention campaigns, (b) GBV government interventions, (c) the importance and perceptions of GBV prevention campaigns. Each theme has subthemes which are backed by the participants’ interview extracts to assist with magnifying the phenomenon investigated. The findings are supported by participant quotations and have been underpinned through the Social Learning Theory and Feminist Theory. The analysis links the participants’ views on weak law enforcement and limited accountability to the reinforcement of violent behaviour, as explained by Social Learning Theory. Similarly, concerns regarding male inclusion, safety, and the protection of women’s voices are interpreted through Feminist Theory and its emphasis on patriarchal power relations. Subsequently, perceptions of ineffective campaigns and rural exclusion are discussed as reflections of broader structural inequalities within GBV prevention efforts, thereby strengthening the study’s theoretical and academic contribution.

**Table 2:** Themes and sub-themes for the study.

Themes	Sub-themes
1. The perceived knowledge of GBV and prevention campaigns	1. GBV as a gendered and power-based violation 2. GBV prevention campaigns as awareness and support strategies
2. Perceived ineffectiveness and failure of government GBV intervention strategies in South Africa	2.1 Ineffectiveness of government GBV interventions strategies 2.2 Justice system failure and normalisation of GBV 2.3 Urban bias and marginalisation of rural communities
3. Evaluating GBV prevention: Importance, limitations, and the need for inclusive approaches 4. The role of governmental and political actors	3.1 The perceived importance of GBV prevention campaigns 3.2 The perceived effectiveness of current GBV prevention initiatives 3.3 Tensions around a multi-gender approach (inclusion of men)

#### Theme 1: The perceived knowledge of GBV and prevention campaigns

The theme focused on exploring the students’ knowledge of GBV, how they define, interpret and make sense of GPV and their understanding of GBV prevention campaigns. Participants provided detailed explanations of GBV and described prevention campaigns as power driven phenomenon embedded in gender inequality and vulnerability thus, reflecting their understanding of the subject. Their responses suggest a clear grasp of GBV and its prevention strategies. Their comments highlighted the importance of GBV prevention initiatives and reinforced the relevance of this study. This suggests that their understanding extends beyond abstract definitions to lived realities of victims, where trust, proximity, and gendered vulnerability shape how GBV is recognised and interpreted.

Subthemes and participants’ responses are detailed.

##### Sub-theme 1.1: GBV as a gendered and power-based violation

Findings in this subtheme indicates that participants had a comprehensive knowledge and awareness of GBV as an act of violence or cruelty which includes physical, sexual, and emotional abuse of victims. Perpetrators of GBV are often identified as men in close relationship with victims. They further highlighted that GBV is a crime against the marginalised vulnerable members of the society such as women, children, and the elderly. Thus, reinforcing dominant gendered narratives of victimisation. This reflects internalisation of broader societal discourses where GBV is closely associated to power imbalance and gender inequality. Below are the participants’ narratives.

*“GBV is an act of violence or cruelty which includes physical, sexual and emotional abuse of victims”. Participant 7*

*“GBV is a crime towards the innocent women, children, and the old people. In most cases the victims are raped beaten or killed in a cruel way by those they trust”. Participant 4*

*“GBV always violates the rights of the vulnerable and is often caused by men closer to the victim”. Participant 6*

##### Sub-theme 1.2: GBV prevention campaigns as awareness and support strategies

Findings uncovered those participants demonstrated a comprehensive understanding of GBV prevention campaigns and their purpose. Furthermore, GBV prevention campaigns are recognise as public education forums on the violence directed towards women and children awareness tool that is geared towards providing a safe space for victims. In addition, GBV prevention campaigns were also seen as victim support, awareness-raising, and social mobilisation initiatives. Of note was that female participants often emphasised survivor empowerment, while their male counterparts stressed awareness and deterrence. The difference in emphasis is shaped by positionality and perceived risk also suggesting that personal vulnerability may shape engagement, with female participants articulating more nuanced understandings related to lived experiences or perceived risk.

*“GBV campaigns are created to educate people on the violence directed towards women and young girls. In South Africa specifically, there is a lot of GBV that the country has been dubbed to be in a war against women. These campaigns are to raise awareness of these issues and curb the spread of this type of violence and create a safe space for victims where they can feel protected.” Participant 5*

*“What I understand about GBV prevention campaigns is that they are put in place to raise awareness on GBV matters, they are there to offer a safe space for GBV victims to raise their voices and ask for help in leaving abusive situations. I think these campaigns are all about the victims instead of the perpetrators” Participant 7*

Generally, understanding GBV prevention programmes requires engagement with feminist theory. Feminism is a philosophy and movement that hopes to eliminate all forms of discrimination against women (McAfee, 2018). By raising awareness of the existing oppressive behaviours, feminist theory aims to address gender inequality in societies (Clark and Horton, 2019). It is only when oppressive behaviours in GBV are recognised that such be combated via actions like activism and prevention campaigns.

#### Theme 2: Perceived ineffectiveness and failure of government GBV intervention strategies in South Africa

This theme focused on exploring the effectiveness and failures of government intervention strategies in preventing and combatting the surge of GBV in SA. The theme further reflects participants’ core views of government efforts, highlighting a lack of trust in institutional responses to GBV.

##### Sub-theme 2.1: ineffectiveness of government GBV interventions strategies

The alarming rate of GBV in South Africa was reported by the participants. Both genders concurred that GBV statistics are still increasing, and more women are still being murdered, even though government-run prevention campaigns have been instituted. Participants expressed mixed perceptions about the government GBV prevention strategies. Only one participant was of the view that the government was putting effort to fight against GBV. However, most participants described government efforts as insufficient, inconsistent, reactive rather than proactive and focusing on high profile cases. These, therefore, concluded that participants viewed the government GBV interventions initiatives a weak in enforcing laws, thus leading to low conviction rates for perpetrators, and poor implementation of policies. Furthermore, inconsistent outreach efforts, under-resourced GBV services, and focusing on high-profile cases at the expense of the ordinary victims added to the ineffectiveness of government GBV interventions initiatives. The perceived ineffectiveness of campaigns reflects a limitation in current interventions. In addition, while Feminist Theory supports awareness as a necessary step, participants’ responses suggest that awareness has not translated into structural transformation. This aligns strongly with Social Learning Theory, which argue that behaviour change requires among other reinforcement, consequences, and observable behavioural models. Lack of these elements may explain the rationale behind the increasing cases of GBV despite high levels of awareness.

*“There are still increasing cases of gender-based violence since the implementation of the campaigns. 2020 was the most tragic one with the most cases of violence, like the incidence of Tshegofatso Pule and others.” Participant 4*

*“People are not made aware enough that when one is experiencing the GBV which steps should they take. More education is needed” Participant 9*

##### Sub-theme 2.2: justice system failure and normalisation of GBV

Participants’ perceptions reflect a broader crisis of institutional trust, where government interventions are not only seen as ineffective but as inconsistent. This shifts the interpretation of GBV prevention from awareness-based efforts to issues of accountability, enforcement, and systemic failure. Participants pointed out that there is a lack of justice for victims and a lack of consequences for perpetrators. They indicated that the statistics of GBV are forever on the rise, the criminal justice system is not doing enough, criminals are not being apprehended, and they receive lesser sentences or are released due to lack of evidence; meaning that justice will not be served for victims. According to Statistics South Africa (2023) and South African Government News Agency (2020) justice failure also result from poorly collected DNA evidence, backlog on forensic processing of specimens, a backlog of court roll, police corruption and untraceable perpetrators. Furthermore, the perceived absence of consequences for perpetrators contributes to the normalisation of GBV, reinforcing participants’ belief that current interventions lack deterrent value. Social Learning Theory supports the argument that perpetrators are emboldened when harmful behaviours are not consistently punished (McLeod, 2016). Unpunished or inadequately punished GBV behaviours are likely to perpetuate and reinforce the behaviour (Bandura, 1977; McLeod, 2016). Furthermore, the reinforcement is also seen in how culture perpetuates the oppression of women (James-Hawkins, 2018). The study also suggests the need for stricter and more consistent enforcement mechanisms that are both punitive and rehabilitative to those who commit acts of GBV. McLeod (2016) also supports the need for visible accountability to deter perpetrators and to help them unlearn harmful behaviours. According to Zuzile (2020) low successful prosecution or conviction rate of the perpetrators was seen as perpetuating GBV, which according to the Feminist theory is the institutional failure to protect the maginalised thus, aggravating gender inequality. The narratives are quoted below:

*“A large number of women are still being killed and the criminal justice system is not doing enough, criminals are not being apprehended, they receive lesser sentences and serious killers are being released because of evidence. Police are not doing their job and are corrupt.” Participant 12*

*“They [government] are making it aware to the perpetrators that such actions will not go unpunished, they let them know that they need to get help and stop the violence they are doing, and the number of violence has started to decrease” Participant 1*

*“The only thing the government does is to sponsor funerals for those whose GBV cases trended on social media, other than that there is nothing that the government is doing to prevent GBV, at least the non-governmental sectors try, but they fail because they do not cater for everyone, in that not everyone is exposed to their services.” Participant 10*

##### Sub-theme 2.3: urban bias and marginalisation of rural communities

The participants also indicates that the government is not doing enough to educate the people about GBV prevention campaigns as such campaigns are urban centred and excludes people in the marginalised rural communities. The participants still reported that the government focuses on cases that attract media interest, high profile cases and leave out the poor people living in the rural townships where GBV is rife. This, therefore, contribute to the increase in GBV cases. Generally, such discrepancy highlights structural inequality, where the most affected rural poor populations are least engaged by institutional support interventions initiatives.

*“The government is not doing anything effective to raise awareness and they are not consistent because femicide is still our daily problem.” Participant 8*

*“There are still increasing cases of gender-based violence since the implementation of the campaigns. 2020 was the most tragic one with the most cases of violence, like the incidence of Tshegofatso Pule and others.” Participant 4*

*“They do not have any impact sometimes. These campaigns only focus on victims instead of also focusing on perpetrators. We only see these organizations being advertised on TV, I've never seen an organization reaching out to people in townships and rural areas, and that's where most GBV cases are, women and children in these places are abused each day and most of them do not know that campaigns are working with these kinds of things, campaigns that offer a safe space for them to talk about GBV and because campaigns don't reach out to these places, perpetrators gain the upper hand because nobody cares about this and the fact that the law drags its feet when it comes to these matters.” Participant 3*

#### Theme 3: Evaluating GBV prevention: importance, limitations, and the need for inclusive approaches

Under this theme four subthemes emerged namely (a) the importance of GBV prevention campaigns (b) the perceived effectiveness of current GBV prevention initiatives (c) tensions around a multi-gender approach (inclusion of men) and the role of non-governmental and political actors. Participants evaluated the GBV prevention campaigns not only in terms of their importance, but also in terms of effectiveness, inclusivity, and sustained impact. Largely, this implies that visibility alone is insufficient, and effectiveness is judged based on whether interventions translate into purposeful social change. The subthemes and the participants’ narratives are outlined below:

##### Sub-theme 3.1: the perceived importance of GBV prevention campaigns

Findings indicate that participants viewed GBV prevention campaigns as necessary in South Africa especially that the rate of GBV against women, children, and the elderly are alarming (Cele, 2021). They indicated that GBV prevention campaigns are safe and supportive platforms created for the victims to share their experiences for the facilitation of psychological healing and support. Furthermore, GBV prevention campaigns were perceived as methods of raising insight and awareness, equipping women with strength, and empowering them with tools to escape abuse. Prevention campaigns also help in the reduction of femicide because awareness enhances victim empowerment that equips victims with skills and resources to help cope with the impact of GBV. In addition, GBV prevention campaigns were also viewed as designed to guide, inform, and make people aware of the dangers and risks of violence. GBV prevention campaigns also help in the reduction of femicide because awareness enhances victim empowerment and create a safe space for victims. The participants’ direct responses are outlined below:

*“Gender-based violence prevention campaigns are campaigns that are designed to guide, inform, and make people aware of the dangers and risks of the violence. They mostly advise or inform people to talk when faced with violence. Leave the most toxic relationships because they are the ones that lead to violent behaviour.” Participant 4*

*“Gender-based violence campaigns are created to educate people on the violence directed towards women and young girls. In South Africa specifically, there is a lot of gender-based violence* that *the country has been dubbed to be in a war against women. These campaigns are to raise awareness of these issues and curb the spread of this type of violence and create a safe space for victims where they can feel protected.” Participant 5*

*“These are campaigns that are responding and protecting the rights of women who are exposed to high risks of any form of violence. They also provide adequate resources to increase the implementation of the programme of awareness specialised for those women. Victims’ rights are prioritised.” Participant 12.*

##### Sub-theme 2: types of GBV prevention campaigns and their limited effectiveness

Participants demonstrated awareness of different types of GBV prevention campaigns and current organisations intended to reduce GBV surge. They identified the three common types of the GBV campaigns namely, the 16 days of activism against GBV, Safer Spaces and POWA (People Opposing Women Abuse, 2018) that are geared towards GBV prevention. However, their effectiveness was found to be limited as evidenced by the rising statistics of GBV cases (Statistics South Africa, 2023) and by the limited behavioural change, especially during specific days earmarked for their awareness. The perceived limitations of current interventions highlight a disconnect between campaign visibility and community-level impact, particularly in rural and marginalised settings. Another participant indicated that they only get to know about those organisations during the television adverts but they never reach out to people in townships and rural areas where most GBV cases are reported. This indicates that the campaigns are visible but not impactful; thus, demonstrating a serious gap between awareness and real-world outcomes. The limited ineffectiveness of these campaigns according to participants is also associated with their victims focus approach instead of roping in perpetrators whom they believe are the force towards change. Furthermore, limited resources and lack of political will strong enough for the government to research a proper national strategic plan to support the fight against GBV and a budget allocation towards GBV prevention and awareness are also implicated in the ineffectiveness of the GBV prevention campaigns. The participants’ narratives are indicated below:

*“Gender-based violence is still rife in South Africa despite campaigns such as Sisonke Gender Justice, 16 days of no violence against women and children, People Opposing Women* Abuse *etc. It is like these crimes are committed more during these specific days”. Participant 8*

*“They do not have any impact sometimes. These campaigns only focus on victims instead of also focusing on perpetrators. We only see these organizations being advertised on TV, I've never seen an organization reaching out to people in townships and rural areas, and that's where most GBV cases are, women and children in these places are abused each day and most of them do not know that campaigns are working with these kinds of things, campaigns that offer a safe space for them to talk about GBV and because campaigns don't reach out to these places, perpetrators gain the upper hand because nobody cares about this and the fact that the law drags its feet when it comes to these matters.” Participant 3*

*“I think that while GBV campaigns are needed and effective, they are not as much as they would be if there was political will strong enough for the government to research a proper national strategic plan to support the fight against GBV and a budget allocation towards GBV prevention and awareness. I think that civil society organisations need a stronger presence and helping hand from the government and in the present moment, the government is not doing enough to show that they are also committed to this fight.” Participant 5*

*“They are not doing anything effective to raise awareness and they are not consistent because GBV is still our daily problem.” Participant 7*

##### Sub-theme 3.3: tensions around a multi-gender approach (inclusion of men) in GBV prevention campaigns

The inclusion of men in GBV prevention campaigns was met with two contradictory viewpoints. Some participants believed men’s involvement is essential in reducing violence and modelling positive masculinity while others feared possible retraumatisation, dilution of women’s voice and making them unsafe in the campaigns. Participants also endorsed that men should be included to better understand the effects of GBV, to make them aware of the impact of their actions on the vulnerable group. Furthermore, including men was viewed as a way of assisting men to better understand the impact of GBV so as to provide support and protection to victims. Some of them felt that men could function as allies in the fight against GBV and therefore viewed men as agents of change. A multi-gender strategy to GBV prevention programmes was seen as crucial in recognising their contribution to GBV.

*“Men should be included to better understand the effects of GBV which will assist in them providing support and protection to potential victims” Participant 3*

*“Yes, because they [Men] are the ones that most perpetuate the violence, more suspects are them and they are the ones that mostly kill women and children. Involving men in the campaigns will make men aware of the impact they are causing and the danger they are instilling in the public” Participant 12*

A clear tension emerged in participants’ views, where support for male inclusion was conditional rather than absolute. While some participants viewed men as necessary allies in transforming harmful norms, others expressed concerns related to safety, trust, and the potential marginalisation of women’s voices. Some female participants showed some resistance against the notion of involving males in GBV prevention campaigns similar to those held by feminist academics and activists (Cornwall and Esplen, 2014). Participants believe that victims’ issues are better understood by victims, others believe that inclusion of men will change the narratives because men are never abused, that GBV is all about women therefore men cannot be trusted with making a difference in this regard. These divergent views suggest that inclusion is not inherently accepted, but rather negotiated within a framework of perceived risk, power, and historical gendered harm. Below are a few participants’ objections to including males in GBV prevention initiatives.

*“If you include men, then it will not be GBV. Men are never abused, married young, or beaten because they are men. The reason it is called GBV is that women are subjected to it while being women.” Participant 2*

*“The inclusion of men in GBV prevention campaigns sometimes agitates the victims and makes them feel unsafe.” Participant 6*

*“Men need to prove themselves worthy and capable of understanding on their own without the help of women. Only when they can be trusted they can be included” Participant 10*

Some female participants expressed strong reservations, stating that before including males in GBV prevention initiatives, men should first demonstrate that they will not “hijack” the women’s course. Their responses conveyed frustration and a strong sense of protectiveness over the movement such as #totalshutdown which centres on female participation in their GBV prevention initiatives (Mulaudzi, 2017). Furthermore, others felt that inclusion of men had more concerns about safety and trust. This concluded that inclusion of men in GBV prevention campaigns was seen as dependent on trust and accountability. Overall, male inclusion was not globally accepted but negotiated within concerns about power, safety, and historical harm. The tension around multi-gender approach poses critical debates within the Feminist Theory, particularly concerns about male dominance, safety, and the potential marginalisation of women’s voices. On the contrary, Social Learning Theory supports the inclusion of men as critical agents of change, as behavioural transformation requires role modelling and the reshaping of social norms, especially among those most likely to perpetrate violence.

#### Theme 4: Role of non-governmental and political actor in the prevention of GBV

##### Contribution of government and social organisations in the prevention of GBV

Participants acknowledged the role played by social organisations, civil society and political parties in the advocacy for the right of the GBV victims while at the same time condemning the lack of robust intervention by the government. They believed that non-governmental entities try their best however, they fail because of lack of resources. Political parties such as EFF were commended for the active support offered to GBV victims. Participants strongly believed that the justice system is corrupt and denies victims justice because of the corrupt police officers who led to the society losing hope in the police. Furthermore, participants were convinced that the government and other political parties only show interest when there is a high-profile GBV case so that they could appear in the news, popular social media platforms and television, newspapers, and radios. Participants’ preference for non-governmental and political organisations reflects a redistribution of trust away from formal institutions towards actors perceived as more responsive and community engaged. The response that follows capture the perceptions of participants regarding contribution of political organisations in GBV prevention campaigns:

*“I only see Economic Freedom Fighters (EFF) doing a change in this type of violence because our justice system is corrupt. My sister helps in GBV along with other comrades and works into getting back the victim or getting justice for the victim.” Participant 12*

*“I hope the EFF grows and helps even more people around our country who are victims of GBV even those who do such acts and justice needs to be served and end corruption in our police stations otherwise we have lost hope in our police.” Participant 3*

*“The only thing the government does is to sponsor funerals for those whose GBV cases trended on social media, other than that there is nothing that the government is doing to prevent GBV, at least the non-governmental sectors try, but they fail because they do not cater for everyone, in that not everyone is exposed to their services.” Participant 11*

## Discussion

The results of this study align with latest universal evidence emphasising that awareness alone is not enough to reduce GBV without corresponding structural and behavioural interventions (Ellsberg et al., 2015; Fulu et al., 2014). Furthermore, Centre for the Study of Violence and Reconciliation (2024) and Statistics South Africa (2024) record that participants’ perceptions of ineffective government responses reflect broader concerns regarding institutional accountability and the implementation gap between policy and practice, a challenge widely documented in South Africa and globally.

Significantly, the conditional support for male inclusion highlighted in this study add to emerging debates within gender-transformative agenda and victim centred protection. While previous research identified the benefits of engaging men as allies (Gibbs et al., 2020; Casey et al., 2023), the results of this study demonstrate that such inclusion is not globally accepted and is shaped by concerns of safety, trust, and power to safeguard women’s voices. This supports debates that male engagement must be carefully structured, balanced, and critically monitored to avoid reinforcing existing gender inequalities (Connell, 2024; True, 2021).

Additionally, the study results extend existing literature by showing that students evaluate GBV prevention campaigns based on perceived legitimacy and community impact rather than visibility alone. This concurs with recent studies indicating that trust in institutions and relevance to local contexts are key determinants of intervention success (Muluneh et al., 2020; Bongalo and Makhubele, 2024). The focus on community-based approaches in participants’ responses further corroborate calls for decentralised and context-sensitive strategies in GBV prevention (World Bank, 2024).

The study further points to context specific awareness by highlighting rural exclusion, perceived urban bias, exclusion of the marginalised communities in interventions, and scepticism towards government effectiveness in the prevention of GBV. Furthermore, the study emphasises that the government and other political parties’ involved is often geared towards advancing their political agendas other than assisting the victims. Significantly, the study pointed out that that participants conceptualised GBV as a gendered and power-based phenomenon, aligning with Feminist Theory, which locates GBV within broader systems of patriarchy and inequality (Hooks, 2000). Just like the findings of True (2021), participants in this study stresses women as primary victims and men as perpetrators which reflects dominant gendered discourses that continue to shape understandings of violence and GBV today.

## Conclusion

GBV remains an ineradicable societal concern in South Africa, which requires comprehensive and context-sensitive interventions. This study highlights that while the inclusion of men in prevention campaigns is generally recognised as critical, it is however, accepted with certain conditional reservations. Instead, it reflects concerns related to safety, trust, and power dynamics by women. The results of this study also accentuate the need for structured, accountable, and survivor-centred approaches (SCA) that balance inclusivity with the protection of the vulnerable groups. Furthermore, results also shift the evaluation of GBV interventions beyond awareness-raising towards the importance of accountability, enforcement, and meaningful behavioural outcomes, particularly in contexts characterised by weak institutional responses. The study further supports a multi-gender approach (MGA) that positions men as accountable associates rather than central actors in the GBV prevention campaigns. Overall, the findings underscore that effective GBV prevention requires context-sensitive, structurally informed strategies that balance inclusivity with the protection and prioritisation of women’s experiences. Furthermore, the study concludes that GBV prevention requires a balanced, multi-sectoral approach that incorporates structural reform with behavioural change strategies; while male inclusion is viewed as potentially beneficial which must be implemented cautiously within feminist-informed, survivor-centred frameworks. In addition, future interventions should prioritise community engagement, institutional accountability, and gender-transformative strategies that address both behavioural and structural drivers of violence. In summary, the study suggests that addressing GBV institutions should gravitate beyond awareness-based strategies toward integrated approaches that combine feminist-informed structural change with behaviour-focused interventions grounded in social learning principles.

## Limitations

This study is limited by its small sample size and focusing on students from a single university, which restricts the diversity of perspectives. Furthermore, the study did not specifically indicate the inclusion of diverse race, class, sexuality, or disability which limited it transferability. The over-representation of female participants may have influenced the findings. Although the findings are not generalisable, they provide context-specific insights. Regarding GBV and inclusion of men in the prevention campaigns. Future research could adopt a more diverse and representative sample, encompassing a wider range of academic backgrounds, demographics, and gender representations.

## Recommendations

The results underscore the need for more inclusive and effective GBV prevention strategies in South Africa. Strengthening community-based interventions is paramount to improve reach within rural and marginalised populations where current campaigns remain limited. The study further highlights the importance of developing structured frameworks for male inclusion, as support for men’s involvement was conditional and dependent on accountability and the protection of survivor-centred spaces. Furthermore, improving institutional accountability is important, given concerns about weak law enforcement, low conviction rates, and systemic inefficiencies. Expanding rural outreach and ensuring equitable access to awareness campaigns and support services are also necessary. Generally, a coordinated, multi-sectoral approach that integrates community engagement, inclusive participation, and institutional reform is required to enhance the effectiveness of GBV prevention efforts.

## Data Availability

The raw data supporting the conclusions of this article will be made available by the authors, without undue reservation.
